# Upregulation of Neural Cell Adhesion Molecule 1 and Excessive Migration of Purkinje Cells in Cerebellar Cortex

**DOI:** 10.3389/fnins.2021.804402

**Published:** 2022-01-21

**Authors:** Shahin Shabanipour, Xiaodan Jiao, Maryam Rahimi-Balaei, Mohamad Reza Aghanoori, Seung H. Chung, Saeid Ghavami, G. Giacomo Consalez, Hassan Marzban

**Affiliations:** ^1^Department of Human Anatomy and Cell Science, Max Rady College of Medicine, Rady Faculty of Health Sciences, University of Manitoba, Winnipeg, MB, Canada; ^2^Department of Pharmacology and Therapeutics, Max Rady College of Medicine, Rady Faculty of Health Sciences, University of Manitoba, Winnipeg, MB, Canada; ^3^Department of Oral Biology, University of Illinois Chicago, Chicago, IL, United States; ^4^Division of Neuroscience, San Raffaele Scientific Institute, Vita-Salute San Raffaele University, Milan, Italy; ^5^The Children’s Hospital Research Institute of Manitoba (CHRIM), Max Rady College of Medicine, Rady Faculty of Health Sciences, University of Manitoba, Winnipeg, MB, Canada

**Keywords:** Bergmann glia, *nax*, neuronal migration, Purkinje cell cluster, mice

## Abstract

Purkinje cells (PCs) are large GABAergic projection neurons of the cerebellar cortex, endowed with elaborate dendrites that receive a multitude of excitatory inputs. Being the only efferent neuron of the cerebellar cortex, PCs project to cerebellar nuclei and control behaviors ranging from movement to cognition and social interaction. Neural cell adhesion molecule 1 (NCAM1) is widely expressed in the embryonic and postnatal development of the brain and plays essential roles in neuronal migration, axon pathfinding and synapse assembly. However, despite its high expression levels in cerebellum, little is known to date regarding the role(s) of NCAM1 in PCs development. Among other aspects, elucidating how the expression of NCAM1 in PCs could impact their postnatal migration would be a significant achievement. We analyzed the Acp2 mutant mouse (*nax*: naked and ataxia), which displays excessive PC migration into the molecular layer, and investigated how the excessive migration of PCs along Bergmann glia could correlate to NCAM1 expression pattern in early postnatal days. Our Western blot and RT-qPCR analysis of the whole cerebellum show that the protein and mRNA of NCAM1 in wild type are not different during PC dispersal from the cluster stage to monolayer formation. However, RT-qPCR analysis from FACS-based isolated PCs shows that Ncam1 is significantly upregulated when PCs fail to align and instead overmigrate into the molecular layer. Our results suggest two alternative interpretations: (1) NCAM1 promotes excessive PC migration along Bergmann glia, or (2) NCAM1 upregulation is an attempt to prevent PCs from invading the molecular layer. If the latter scenario proves true, NCAM1 may play a key role in PC monolayer formation.

## Introduction

Neuronal migration and positioning are critical steps of development mediated by several cellular and molecular interactions that promote the assembly of neuronal circuits, a process that is fundamental for brain function (Rahimi-Balaei et al., 2018). The cerebellum is critical for motor control and cognitive function and is comprised of a few distinct neuronal subtypes. The cerebellar three-layer cortex consists of the molecular layer, Purkinje cells (PC) layer, and granule cell layer. The main cells in the PC layer are a single row of PC somata, which are intermingled with bodies of Bergmann glial cells (BGCs). PCs are the sole output neuron of the cerebellar cortex and are arranged in an elaborate monolayer throughout the entire cerebellar cortex (Voogd, 1992).

During embryonic development, the cerebellar primordium contains two distinct germinal zones: the ventricular zone and the rhombic lip (Englund et al., 2006; Fink et al., 2006). PCs are derived from the ventricular zone, and complete their final mitotic division at E10–13 in mice (Miale and Sidman, 1961; Wang and Zoghbi, 2001; Marzban et al., 2014). At the post-mitotic stage, PCs start migrating a short distance from their site of origin in the ventricular zone and accumulate in the PC Plate. The migration of PCs from the ventricular zone is described as the glial-guided as PCs migrate along radial glial fibres during early embryonic cerebellar development (Li et al., 2014; Sergaki and Ibanez, 2017; Rahimi-Balaei et al., 2018; Schilling, 2018). PCs eventually change their position and reside as a cluster in the cerebellum during perinatal development. Finally, a new wave of PC migration initiates at around postnatal day (P)2, during which the PC clusters disperse and finally line up in the PC monolayer by P7 (Rahimi-Balaei et al., 2018).

At P2, the BGC bodies colocalize with the PCs in the cluster. BGCs are astrocytes originated from radial glia progenitors (Das, 1976). The movement of BGCs in the developing cerebellum at around P2 coincides with PC dispersal. By P7, both cells ultimately reside in a linear shape conformation called the PC layer. Several studies have shown that BGCs play a crucial role in regulating the movement of granule cell progenitors from the external germinal zone (EGZ) to the granular layer. BGCs have been suggested to share similar interactions with other neurons such as PCs and promote their migration (Higuera et al., 2017). Therefore, the PCs-BGCs intercellular crosstalk at P2-P7, is a likely prerequisite for a coupled migration and for the ability to form a definitive PC layer (Rahimi-Balaei et al., 2018).

Further studies also suggested the presence of intracellular junctions between BGCs and PCs (Bellamy, 2006; Elias et al., 2007; Famulski et al., 2010) that with variation would effectively set a specific spatiotemporal pattern for migration of both cells. It has been shown that the interactions between neuron, glia and extracellular matrix (ECM) would impact not only the migration of PCs but also the function of neural cell adhesion molecule 1 (NCAM1) which is one of the most typical member of cell adhesion molecules of the immunoglobulin superfamily (IgSF CAMs) (Mendis et al., 1994; Kearns et al., 2003; Sergaki and Ibanez, 2017). NCAM1 plays essential roles in migration of precursor cells and pathfinding of axons (Maar et al., 1998; Huang et al., 2006; Stoeckli, 2010). Studies on NCAM1 deficient mice and cerebellar explants showed that the lack of NCAM1 in PCs enhances their migration in the embryonic cerebellum (Rakic and Komuro, 1995; Schmid and Maness, 2008; Sergaki and Ibanez, 2017). PSA (polysialic acid) -NCAM1 interactions direct migration and differentiation of neural precursors during development (Schmid and Maness, 2008; Sergaki and Ibanez, 2017).

In this study, we investigated the expression pattern of NCAM1 in the whole cerebellum and the isolated PCs and BGCs during the PC dispersal period. For this purpose, *Acp2* (acid phosphatase 2, lysosomal) mutant mice (aka; *nax*, naked and ataxia) exhibiting excessive PCs migration were examined and the transcription of *Ncam1* was evaluated. Our results show a significant increase in *Ncam1* transcription in PCs and BGCs of the *nax* cerebellum. *Nax* PCs express abnormally high levels of *Ncam1* and yet feature excessive migration, while, in the wild type cerebellum, low *Ncam1* expression levels are sufficient to arrest PCs migration at the PC layer (Sergaki and Ibanez, 2017). BGCs also upregulate *Ncam1* in the *nax* cerebellum. Our data suggest that NCAM1 may be responsible for fine-tuning PC migration and alignment in the cerebellum. The potential role of *Ncam1* in the context of PC migration will be discussed below.

## Materials and Methods

### Animal Protocol

In this study, the naked ataxia (*nax*; *Acp2 −/−*) mice were used for investigating abnormal PC and BGC positioning in the cerebellar cortex. All the animal experiments were submitted and approved by institutional regulations and *the Guide to the Care and Use of Experimental Animals* from the Canadian Council for Animal Care (CCAC). This study was approved by the University of Manitoba Animal Care Committee (ACC) and efforts were made to minimize the number of animals that we needed for our experiments. Immunohistochemistry, Western blotting and RT-qPCR (tissue and sorted cells) examination were performed at least in three animal trials per each age (P2 and P7).

### Animal

*Acp2 mutant mice (nax mice)* were obtained by importing the *nax* mutant embryos from the Institute of Human Genetics in the University Medical Center, Georg-August University, Gottingen, Germany followed by establishing the colony in the Genetic model center of University of Manitoba (Bailey et al., 2014). An average of 3–4 mice from each strain were housed per home cage under standard 12 h light/dark cycle. For immunohistochemistry experiment, the perfusion of mice was carried out with 4% PFA, after which the brain was removed from the skull and immersed in the same fixing solution. The brain tissue was washed with 1×PBS (phosphate-buffered saline) and frozen embedded in OCT. Using the cryotome, 25 μm-thick tissue sections were obtained.

### Immunohistochemistry

To demonstrate the expression pattern of the NCAM1 in developing cerebellum at P2 and P7, brain tissue sections were processed through immunohistochemistry and co-labeled with PCs, BGCs markers and the NCAM1. Cerebella of at least three mice for each strain were collected and at least seven sections from cerebellar vermis were analyzed in each animal. During immunohistochemistry process, the tissues sections were blocked with NGS (Normal goat serum 10% including 0.3% triton × 100) for an hour at room temperature (RT) and then incubated with the following primary antibodies: Rabbit polyclonal anti-calbindin D-28K antiserum CALB1 (Swant Cat# CB38, RRID:AB_10000340, 1:5,000), mouse monoclonal anti-S-100 (β-Subunit) antibody (Sigma-Aldrich Cat# S2532, RRID:AB_477499, 1:500) and mouse monoclonal_anti-NCAM1 (DSHB Cat# 5b8, RRID:AB_528393, 1:100) overnight at 4°C. After washing with PBS 1×, the sections were incubated 1 h with secondary antibodies: Polyclonal Alexa 488-conjugated goat-anti-mouse IgG (Thermo Fisher Scientific Cat# A-11029, RRID:AB_2534088 1:1,000) and Polyclonal Alexa 549-conjugated goat-anti-mouse IgG (Thermo Fisher Scientific Cat# A-11012, RRID:AB_2534079 1:1,000). The florescent labeled sections were mounted with Fluor Save Reagent, Fluorsave (Calbiochem: Cat# 345789) and visualized with Zeiss Light Sheet Z.1 microscope (Zeiss, Toronto, ON, Canada) equipped with a camera. The imaging of the immunostained sections from each group were carried out under similar lightening conditions. Adobe Photoshop CS5 Version 12 was used to edit, crop, and correct contrast and brightness of the images.

### Western Blotting

The protein analysis of NCAM1 using Western blot was carried out on both *nax* and *wt* samples comprised of 3 cerebella for each strain. The collected samples were covered with lysis buffer (composed of protease inhibitor cocktail (Life Science, Cat# M250) and phosphatase inhibitor (Sigma Aldrich, Cat# P5726)) and homogenized by sonication. For immunostaining, membranes were blocked for 1 h in 5% skim milk in TBS + 0.1% Triton X-100 (TBST). Membranes were incubated with the mouse monoclonal anti-NCAM1 (DSHB Cat# 5b8, RRID:AB_528393, 1:100) primary antibody at 4°C overnight with gentle agitation. Blots were washed with PBS 1× and incubated with secondary antibodies HRP conjugated polyclonal goat anti-mouse IgG (Millipore Cat# AP308P, RRID:AB_92635, 1:7,500) and developed with Clarity Western ECL Substrate (Bio-Rad, Cat# 170506).

### Fluorescence Activated Cell Sorting (FACS) of Purkinje Cells and Bergmann Glial Cells

Mice were selected from both *wt* and *nax* strains each at two different times (P2 and P7). At the day of the experiment, mice were anesthetized, and the dissected brains were immediately transferred into ice-cold 1× Hank’s balanced salt solution (HBSS, Gibco 14185-052) and washed by changing the buffer 3×. Afterward, tissues were transferred to the dissection medium (1× HBSS containing gentamicin 10 μg/mL) for cerebellum isolation.

Following the cerebella isolation, washing steps of the collected samples were carried out (Centrifuge at 300 *g*, 4°C and change supernatant) in cold Dulbecco’s modified Eagle medium: nutrient mixture F-12 (DMEM/F12, Lonza 12-719F) 3× 1 min, and then incubated in trypsin (Gibco 15090-046) (37°C) for about 12 min. The trypsin was inactivated with 10% FBS (fetal bovine serum) – DMEM/F12 media and washed 3× 5 min.

The pellets were mildly triturated with the 3.5 mL of DNase working solution (1 mL of DNase I stock solution [0.05% DNase (Roche 11284932001) + 12 mM MgSO_4_ + 1 × HBSS] in 500 μL of heat-inactivated FBS and 2 mL of DMEM/F12) to get a homogenous mixture of cells as a result.

The collected cells were counted (1 × 10^6^) and blocked with NGS 10% without triton X100 at RT for 15 min. The cells were then incubated at RT for 30 in primary antibodies: Mouse monoclonal anti- Kirrel2/NEPH3 (R and D Systems Cat# MAB2564, RRID:AB_2130844, 1:200) for PCs and Rabbit polyclonal anti- EAAT1/GLAST-1/SLC1A3 (Novus Cat# NB100-1869SS, 1:200) for BGC, and afterward were washed 3× with staining buffer. Followed by incubation with secondary antibodies: Polyclonal Alexa488-conjugated goat-anti-rabbit IgG (Thermo Fisher Scientific Cat# A32728, RRID:AB_2633277, 1:500) and Polyclonal Alexa 647-conjugated goat-anti-mouse IgG (Life Technologies, Catalog No., 1:500) for 30 min and counterstaining with DAPI, the final pellet of cells was re-suspended in staining buffer. The staining buffer (1× PBS, FBS 1%, 25 mM HEPES, 1 mM EDTA) was used for diluting antibodies and washing steps during immunohistochemistry. Cell sorting was performed on BD FACS Aria-III cell sorter. Cells were sorted with 100-micron nozzle at the concentration of 15 million cells per ml in purity mode. PCs and BGCs were collected in 15 ml Falcon tubes without any buffer. The next step after sorting the cells would be immediate RNA extraction. Data were acquired on a CytoFlex-LX flow cytometer (Beckman Coulter) equipped with 355, 375, 405, 488, 561, 638, and 808 Laser lines using the CytExpert software, and analyzed with Flow Jo software (version 10) (Treestar, San Carlos, CA, United States) at the University of Manitoba flow cytometry core facility. Cellular debris was excluded using forward light scatter/side scatter plot.

### RNA Extraction From Sorted Purkinje Cells and Bergmann Glial Cells and RT-qPCR Analysis

The average number of sorted cells per each sample that were used for RNA extraction were about 50 × 10^3^. Sorted cells were collected in tubes with the least volume of PBS 1×. After a short spin, the supernatant was removed and immediately the lysis buffer from Qiagen RNeasy Plus Mini Kit was added to the pellet. The rest of the steps were followed according to the instruction of the kit to have the high yield of RNA extraction and avoid contaminations as much as possible (QIAGEN: Cat#/ID: 74134). The cDNA synthesis was performed by 0.25 ng of RNA in a qScript cDNA SuperMix kit (Cat# 95048-100). The reaction mixture contained 10 μL PowerUp™ SYBR™ Green Master Mix (Cat# A25742), 2 μL cDNA template and 1.2 μL of each forward and reverse primers in a total reaction volume of 20 μl. Thermocycling parameters were: 95°C for 3 min followed by 40 cycles of 95°C for 30 s, 55°C for 60 s, 72°C for 60 s. The primer sequences are NCAM1 forward: 5′-TGGTTCCGAGATGGTCAGTT-3′ and NCAM1 reverse: 5′-GGATGGAGAAGACGGTGTGT-3′, GAPDH forward: 5′-GGTGAAGGTCGGTGTGAACG-3′ and GAPDH reverse: 5′-CTCGCTCCTGGAAGATGGTG-3′. All reactions were performed in duplicate, and gene expression values were normalized with respect to the reference gene, GAPDH, and utilizing the 2^–ΔΔ*ct*^ method. Data are presented as means ± SEM.

### RNA Isolation and RNA Sequencing of *nax* and *wt* Cerebellum

The cerebella of *nax* (P5; *n* = 2 and P7; *n* = 3) and *wt* littermates (P5; *n* = 3 and P7; *n* = 3) were isolated and RNA extraction was carried using the RNeasy Plus Mini Kit (Cat# 74134, QIAGEN, Toronto, ON, Canada). The RNA concentration of the collected samples was measured by Nano-Drop ND-1000 UV-Vis Spectrophotometer (Thermo Fisher Scientific, Waltham, MA, United States). The samples were frozen in −80 and sent to the McGill University and Genome Quebec Innovation Centre (MUGQIC) for RNA sequencing. The outcome analyzing (Jiao et al., 2021), raw data from RNA sequencing was the “Reads Per Kilobase of transcript, per Million mapped reads” (RPKM) calculated for each gene.

### Statistical Methods

All experiments were repeated at least 3 times per each selected age of the strain. The raw quantitative (Western blot and RT-qPCR) results of each gene were then analyzed based on comparing the two variances of time point and strain in ANOVA. The analysis and figure preparations were carried out using ANOVA multiple comparisons testing in Prism GraphPad V7.05 and the *p* ≤ 0.05 was considered significant.

## Results

### Excessive Purkinje Cell Migration and Bergmann Glial Cell Positioning in the *nax* Cerebellar Cortex

**FIGURE 1:**
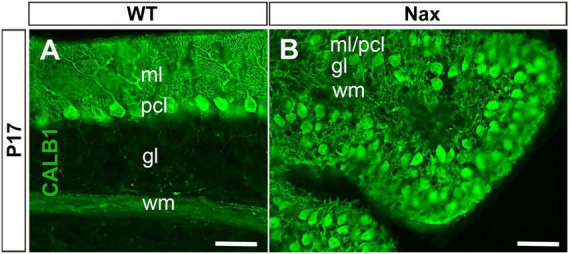
Sagittal sections at P17 of the *wt* and *nax* cerebellum. **(A)** Immunostaining with anti-calbindin (CALB1) in a section through the cerebellum of the *wt* sibling showing the dendrites of PCs in the molecular layer (ml) and Purkinje cell (PC) soma in the Purkinje cell layer (PCl). **(B)** In the *nax* cerebellum showing that the PCs fail to form a uniform monolayer and PCs soma are intermingled in the ml; labeled as pcl/ml. gl, Granular layer; wm, white matter. Scale bar: *A* = 50 μm; *B* = 50 μm.

**FIGURE 2:**
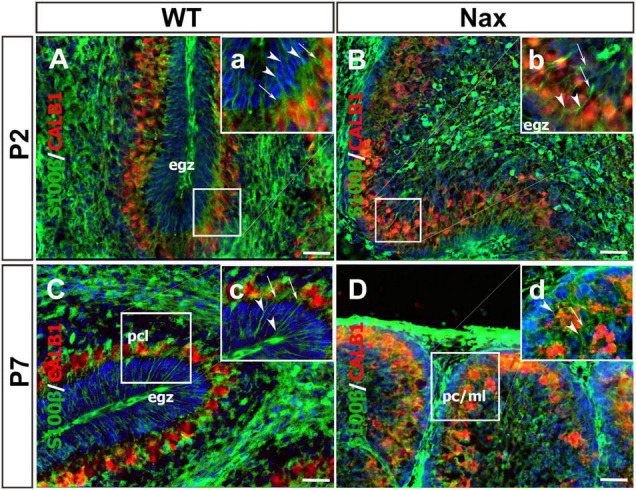
Close relation of BGCs and PCs at P2 and P7 in *wt* and *nax* littermate cerebellum. **(A–D)** Sagittal section of the cerebellum at P2, and P7 were immunofluorescence labeled with anti-calbindin (red) (CALB1; specific marker for PCs) and anti-S100β (green) (specific marker of BGCs). **(A,B)** Images show multiple layers of PCs and the BGC soma at P2 in both *wt* **(A)** and *nax* **(B)** cerebellum. The BGC fibers (arrowhead in high magnifications **a,b**) are extended from basal end and pass the cluster of PCs’ (arrow in high magnifications **a,b**) intercellular space where BGC soma is located. In other word, the extension ends in pial surface and the BGC soma are located in PCs cluster. **(C,D)** The location of BGCs (arrowhead in high magnifications **a,b**) is shown at P7 among PCs in PC layer of *wt* cerebellum **(C)**. BGCs and their fibers are located in *nax* cerebellar sections in PC/molecular layer close to EGZ **(D)**. The BGC fibers in *nax* cerebellum are not well organised as in *wt* cerebellum, but still basal end are connected to the pia matter through their fiber extensions. PCs, Purkinje cells; BGCs, Bergman glia cells; pcl, Purkinje cell layer; ml, Molecular layer. Scale bars: 100 μm (applies to panels **A–D**); 50 μm (applied to panels **a–d**).

To demonstrate the close relationship of PCs and BGCs during postnatal PCs migration, we used the *nax* cerebellum, which features excessive PC migration in the molecular layer. Multilayered PCs in the molecular layer of the *nax* cerebellum at P17, immunostained with anti-calbindin (Calb1) indicate an impairment of PC cluster dispersal to form monolayer during early postnatal development (Figures 1A,B; Bailey et al., 2014). To visualize the arrangement of PCs and BGCs, the cerebellar cortex was immunostained by anti-Calb1 and S100B, respectively, at P2, corresponding to the PC cluster stage, and P7, when PCs are dispersed and organized as a monolayer. In the mouse cerebellum, Calb1 is a specific marker of PCs (e.g., Baimbridge and Miller, 1982; Shabanipour et al., 2019), while S100B was used as a marker of both mature BGCs and their precursors (Raponi et al., 2007; Koirala and Corfas, 2010). At P2, a double immunofluorescence staining with anti- Calb1 and anti- S100B in both the *wt* (Figure 2A) and *nax* (Figure 2B) cerebellum shows that PCs form a multicellular cluster. The S100B immunopositive BGC somata (Figures 2A,a,2B,b; arrow) at this point colocalize with PC bodies, while their fibers extend toward pial surface in both the *wt* (Figures 2A,a; arrowhead) and *nax* (Figures 2B,b; arrowhead) cerebellum. At P7, in the *wt* cerebellum, PCs are found in the PC layer and are accompanied by BGC somata with their fiber extending into the EGZ (Figures 2C,c; higher magnification in the inset). In contrast, in the *nax* cerebellum, PCs are arranged in a multilayer that invades the molecular layer, and fail to align in a monolayer (Figures 2D,d; higher magnification in the inset). Despite the impairment in migration and positioning, PC somata are still intermingling with BGC (Figures 2D,d; arrow). The colocalization of PCs and BGC even when PCs become ectopic (i.e., excessive migration to the molecular layer in *nax* cerebellum) may suggest the existence of cell-cell interactions during migration.

### Neural Cell Adhesion Molecule 1 Expression Pattern in the Cerebellar Cortex

**FIGURE 3:**
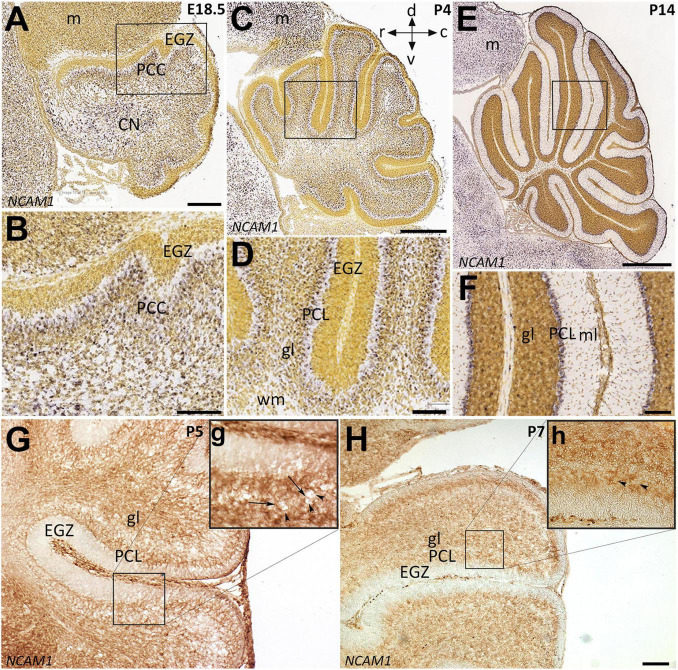
Distribution of NCAM1 in sagittal sections hybridized *in situ* with antisense riboprobes specific for NCAM1 and immunoperoxidase staining in the cerebellum during development. Positive territories are labeled black. **(A–D)** NCAM1 expression at E18.5 and P4 is localized generally in whole cerebellum mostly in PC cluster and CN, except in EGZ and VZ (E18.5; **A** and P4; **C**, and higher magnification in **B,D**). **(E,F)** At P14, NCAM1 expression is localized in PC layer and clearly in PC soma (**E**, and higher magnification in **F**). Image credit: Allen Institute. © 2008 Allen Institute for Brain Science. Allen Developing Mouse Brain Atlas. Available online at: https://developingmouse.brain-map.org/. **(G,H)** Sagittal sections of P5 and P7 *wt* mouse cerebella immunostained with NCAM1 show the expression of NCAM1 is distributed in the granular layer and in PC layer, but not in EGZ, higher magnification provided in **(g,h)**. CN, cerebellar nuclei; EGZ, external germinal zone; gl, granular layer; m, mesencephalon; ml, molecular layer, NTZ, nuclear transitory zone; PCL, Purkinje cell layer; PCP, Purkinje cell plate; RL, upper rhombic lip; VZ, ventricular zone; WM, white matter. Scale Bars = *A* = 198 μm (applies to panels **A–F**); *H* = 100 μm (applies to panels **G,H**).

Neural cell adhesion molecule 1 is a member of the IgCAM (immunoglobulin cell adhesion molecule) superfamily broadly expressed in the neuroglia network not only during embryonic development but also in adults (Reyes et al., 1991; Huang et al., 2006; Magdaleno et al., 2006). To determine the distribution of the *NCAM1* in the cerebellum during PCs monolayer formation, we used *in situ* hybridization data from “Allen Developing Mouse Brain Atlas” [Image credit: Allen Institute.] and, in parallel, performed immunoperoxidase staining in cerebellar sections. The *Ncam1* expression pattern in sagittal sections during cerebellar development showed dynamic expression from E18 to P14. At E18 and P4, *Ncam1* is expressed in the PCs cluster region with low abundance in the EGZ (Figures 3A–D). At P14, NCAM1 expression is clearly localized in the PC layer, with scattered immunoreactivity in the granular layer (Figures 3E,F). Immunoperoxidase staining at P5 and P7 shows a similar pattern, as revealed by *in situ* hybridization, as well as localization in the PC layer (Figures 3G,g,H,h).

**FIGURE 4:**
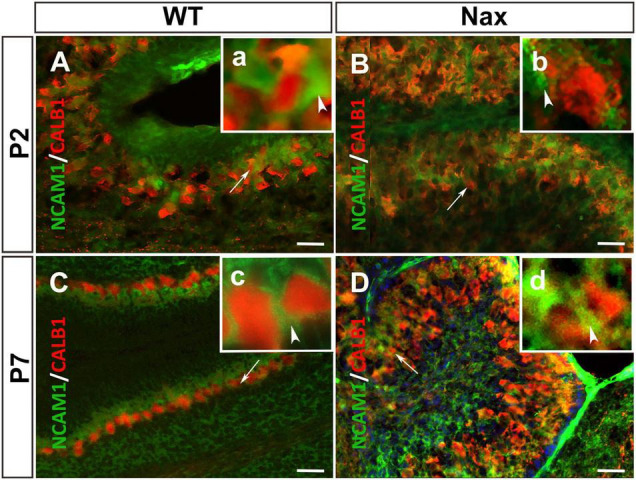
PCs and the expression of NCAM1 in *wt* and *nax* cerebellar cortex. **(A,B)** Sagittal sections through the cerebellum immunostained with anti- calbindin (red) and anti-NCAM1 (green) shows the colocalization of PCs (red arrowhead in higher magnification **a**) and NCAM1 (arrowhead in higher magnification **b**) at P2 in both *wt* **(A)** and *nax* **(B)** cerebellum. **(C,D)** Images at P7 similarly shows the intercellular expression of NCAM1 not only in PC layer of *wt* **(C)** cerebellum but also in *nax* **(D)** cerebellum where the multilayer PCs are invaded into the molecular layer; **(c,d)** Higher magnification of the local NCAM1 expression. PCs, Purkinje cells. Scale bars: *A* = 50 μm (applies to panels **A–D**).

To examine whether NCAM1 colocalizes with PCs, cerebellar sections were double-stained using anti-NCAM1 and anti-Calb1. At P2, *NCAM1* expression is localized in the PCs cluster region of *wt* (Figures 4A,a; arrowhead) and *nax* cerebellum (Figures 4B,b; white arrowhead) highlighting the intercellular spaces. Similarly, at P7, *NCAM1* is expressed in the PC layer in the *wt* (Figures 4C,c) while in the *nax* cerebellum it is found in the area where the multilayered PCs are located, including the molecular layer (Figures 4D,d; arrow). NCAM1 immunoreactivity is also strong in the leptomeninges of the *nax* cerebellum (Figure 4D).

### Neural Cell Adhesion Molecule 1 Expression Patterns Are Altered in the Purkinje Cells and Bergmann Glial Cells of *nax* Cerebellum

**FIGURE 5:**
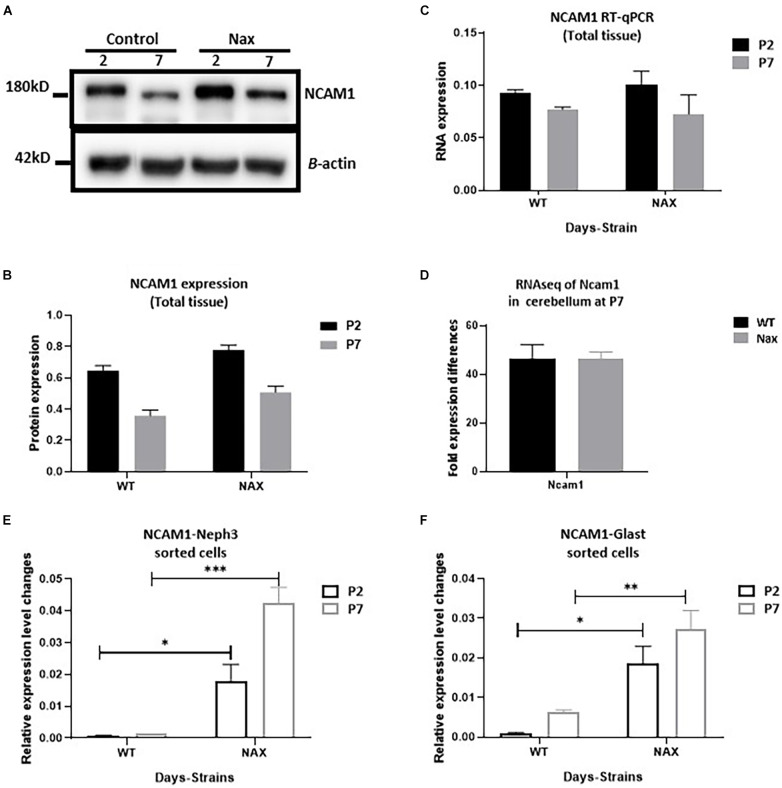
Transcription of NCAM1 in *nax* PCs and BGCs significantly increases. **(A–C)** NCAM1 expression and transcription in the whole cerebellum tissue is measured by Western blot **(A,B)** and RT-qPCR **(C)** at P2 and P7. The NCAM1 protein and mRNA values does not show significant difference between *wt* and *nax* cerebellum (blot image of P2, P5, and P7 in Supplementary Figure 1). **(D)** RNAseq analysis of *nax* and wt cerebellum at P5/P7 to confirm total cerebellum RT-qPCR results; Ncam1 transcription shows the same value in both *nax* and *wt* cerebellum. **(E,F)** RT-qPCR of RNA collected from isolated PCs and BGCs from *nax* and *wt* cerebellum at P2 and P7. The mRNA value of NCAM1 in *nax* cerebellum from both P2 and P7 is significantly higher than the *wt* littermate. This experiment was repeated over three different litters for each postnatal day in *nax* and *wt* littermate (*wt*; *n* = 3 and *nax*; *n* = 3). The data in the bar graph are presented as the mean ± SEM, and statistical analysis was performed using multiple comparison ANOVA (*p < 0.05, **p < 0.01, ***p < 0.001). PCs, Purkinje cells; BGCs, Bergman glia cells. Scale bars: *A* = 50 μm (applies to panels **A–D**).

To examine the NCAM1 protein expression pattern during postnatal cerebellar development, Western blotting was carried out using anti-NCAM1 antibody on *wt* and *nax* cerebella at P2 and P7 (Figure 5A). NCAM1 expression did not change by age between P2 and P7 in either the *nax* or *wt* mice cerebellum. Moreover, the expression levels of *NCAM1* were not significantly different between *nax* and *wt* littermate cerebella at P2 and P7 (Figure 5B and Supplementary Figure 1). In order to elucidate the correlation between NCAM1 protein and mRNA data, total *Ncam1* RNA of cerebellum was analyzed with RT-qPCR as well (Figure 5C) and the results were compared to the collected NCAM1 data. Although the total mRNA results did not reach statistical significance, their levels also showed a downward trend at P2 to P7. RNA-seq data was shown no significant differences in the expression of Ncam1 between wt and nax littermates (Figure 5D).

To better characterize *Ncam1* mRNA levels in isolated PCs and BGCs, we used FACS. To this end, PCs and BGCs were isolated from the cerebellum at P2 and P7, both in *nax* and *wt* littermates (Supplementary Figure 2), and RNA was extracted for RT-qPCR analysis. Results showed that *Ncam1* mRNA levels in both PCs (Figure 5E) and BGCs (Figure 5F) are substantially enhanced in the *nax* cerebellum as compared to the *wt* cerebellum (*p < 0. 05, ^**^p < 0.01, ^***^p < 0.001), a difference that was not revealed by the analysis of total lysates. The observed increase of *Ncam1* levels in *nax* PCs and BGCs may underlie their excessive migration toward the pial surface and failure of proper alignment into a mature PC layer.

## Discussion

The PC layer consists of a single row of PC somata, which are intermingled with BGC bodies. PCs complete their final mitotic division at E10–13 in mice (Miale and Sidman, 1961; Marzban et al., 2014). At the post-mitotic stage, PCs start migrating a short distance from their site of origin in the ventricular zone and accumulate in the PC plate. Then, clustered PCs initiate their dispersal at around P2 to establish the PC monolayer by P7 (Rahimi-Balaei et al., 2018). Several studies have shown the interdependency of PCs and BGCs for their origins during embryonic development, and continued differentiation until postnatal days has been reported (Fisher, 1984; Bellamy, 2006). During prenatal cerebellar development, the migration of PCs is suggested to be guided by radial glial fibres and the interaction between these two cell types is mediated by cell adhesion molecules, including NCAM1 (Yuasa et al., 1991; Sergaki and Ibanez, 2017).

In the present study, we describe the effects of the *Acp2* gene mutation in the *nax* cerebellum, in which PCs with excessive migration invade the molecular layer (Bailey et al., 2014; Rahimi Balaei et al., 2016). Immunostaining shows that PC somata and BGCs bodies are attached together; in the normal cerebellum (coupled in the PC layer), and in the *nax* cerebellum (in the molecular layer, which is invaded by PCs due to excessive migration). In addition, NCAM1 expression shows adhesion between PCs and BGC in the *wt* PC layer and in *nax* cerebellum’s molecular layer.

The protein and mRNA analysis of NCAM1 in total cerebellum from both *nax* and *wt* mice does not show a significant difference between *strains* and ages (P2 and P7). In contrast, RT-PCR results from sorted cells show that NCAM1 expression in both PCs and BGCs of the *nax* cerebellum is significantly higher than *wt* littermate cerebellum. Comparing the RT-qPCR results from total tissue to the sorted cells counterpart may suggest the following:

1. The majority of NCAM1 expression in *wt* cerebellum takes place in cells other than PCs and BGCs. This may reflect the high requirement of many cells for this adhesion molecule during cerebella development and PC dispersal.
2. The *wt* PCs and BGCs normally have low NCAM1 expression levels. *In situ* hybridization results reveal that *Ncam1* mRNA is detected in *wt* PCs not only at embryonic stages (E18) but also around PC layer formation and even weeks after PCs migration has completed (P14). Moreover, mRNA analysis of sorted cells shows low expression of NCAM1 in PCs and BGCs of *wt* cerebellum. It is well established that the cell–matrix or cell–cell adhesions can be modulated through a change in NCAM1 expression (Gorain et al., 2019). If there is any NCAM1 mediated interaction to be established between PCs and BGCs/ECM and start a new wave of migration, low expression of NCAM1 at P2 is reasonable. Maintaining the low expression of NCAM1 in PCs might be the proper way to tune the cell migration until PCs are settled in the PC layer. In *wt* BGCs, however, NCAM1 transcription increases at P7. It is believed that this increase, would accelerate the NCAM1 interactions with ECM components such as integrin and prevent further movement of PCs from PC layer. In line with these results, several *in vitro* and *in vivo* studies have suggested that NCAM1 expression inhibits the migration of glioma cells (Edvardsen et al., 1994; Gratsa et al., 1997).

It is also well known that the ECM of the cerebellar cortex is enriched with laminin and collagen regions (Sur et al., 2014). The interaction of NCAM and Integrin β-1 with laminin might increase cell motility (Miyamoto et al., 1995; Tulla et al., 2008). In our studies, the RT-qPCR analysis of NCAM1 in *nax* cerebellum suggests that the post-mutation upregulation of NCAM1 expression in BGCs may trigger the NCAM1-integrin interaction and promote the migration of BGCs toward the pial surface. Our RNAseq data shows a significant increase in mRNA expression of Itgα3 and Itgα5 in the whole *nax* cerebellum at P5/P7 compared to the *wt* littermate (Supplementary Figure 3). Increasing the expression of integrins at P5/P7 may promote cells migration for *nax* PCs. Unlike integrins, increasing the NCAM1 mRNA level in *nax* PCs compared to *wt* might be an unsuccessful attempt to compensate for PCs failing to stop at the PC layer. This raises the possibility that changing ECM components at P7 in the *nax* cerebellar cortex is the main reason of initiating NCAM1 compensatory mechanism and PCs excessive migration. Other studies showed that removing PSA from NCAM1 in PCs can adversely affect their migration (Sergaki and Ibanez, 2017). It is possible that both *nax* PCs and BGCs respond to removing PSA from NCAM1 by upregulating NCAM1 expression in these cells. Our study shows that the connection of PCs and BGCs during postnatal migration in cerebellum needs to be further investigated.

Currently, it is believed that the position of PC cluster changes along with cerebellar surface development, which is extended rostrocaudally and mediolaterally and arranged in a monolayer because of cerebellar expansion (Butts et al., 2014). It is also suggested that granule cells are major players in the migration and positioning of PCs postnatally (Jensen et al., 2002). However, several reports have shown that despite granule cells defects (such as hypoplasia and agenesis), PCs respond differently to these anomalies. In most cases, each affected cerebellum comprises several small percentages of PCs population, which are either in different ectopic locations or arranged in monolayer positions in cerebellar cortex. For example, three populations of ectopic PCs were described in a study of the Atoh1 null-mutant mouse, in which the external granular layer does not form (Jensen et al., 2002). In addition, the scrambler (mutation in Dab1, Reelin adaptor protein) cerebellum is small because the size of the granule cell population is severely decreased by ∼80% and around 95% of PCs (not all) fail to complete their migration (Goldowitz et al., 1997; Reeber et al., 2013). Furthermore, Cxcr4 (a chemokine receptor) deficiency results in fewer granule cells in the cerebellum and partially disorganized ectopic PCs (Huang et al., 2014), which is almost similar to the phenotype described in Weaver mouse (Chen et al., 2009). It has been suggested that protein tyrosine phosphatase, non-receptor type 11 (Ptpn11) regulates formation of the laminar cerebellar cortex by controlling granule cell migration via mediating Cxcl12/Cxcr4 signaling (Hagihara et al., 2009). However, removing Ptpn11 in the external granular layer has no distinct effect on cerebellar corticogenesis (Li et al., 2014). Despite the important role of granule cells, they are not the main player in PCs migration and organization during cerebellar postnatal development. Therefore, such an elaborate PC monolayer organization cannot be explained by surface expansion and granule cells development alone, and this strongly indicates a phenomenon that is precisely regulated by an active cellular and molecular process rather than by passive expansion. Our study suggests that PCs move under molecular changes and regulations assisted BGCs to establish elaborated PC monolayer organization. In particular, our results indicate that NCAM1 in PCs and BGCs may function as a cell movement tuning mechanism that is required at early postnatal days of PCs migration. Finally, our results may also suggest that other molecules such as integrins (Itgα3 and Itgα5) in the ECM, may contribute to NCAM1 upregulation in *nax* PCs and BGCs and promote excessive PCs migration at P7.

## Conclusion

In this study, we describe the spatiotemporal distribution of NCAM1 in PCs and BGCs during early postnatal development. We showed that the relationship between PCs and BGCs is greatly affected by gene mutation (*ACP2−/−*) and time. The close relationship between PCs and BGCs causes these two cell types to stay tightly coupled during their postnatal migration. In the *nax* cerebellum, changes in BGC position toward molecular layer are accompanied by excessive PCs migration. NCAM1 expression pattern in the *nax* cerebellum fits the criteria for excessive coupled migration of PCs and BGCs. NCAM1 overexpression in PCs and BGCs may increase the cell-ECM connections and drag the cells to an ectopic location toward pial surface and suggest that NCAM1 is an essential regulator during PCs dispersal and monolayer formation.

## Data Availability Statement

The original contributions presented in the study are included in the article/Supplementary Material, further inquiries can be directed to the corresponding author/s.

## Ethics Statement

The animal study was reviewed and approved by the study was conducted according to the guidelines of the institutional regulations and the Guide to the Care and Use of Experimental Animals from the Canadian Council on Animal Care, and approved by local authorities “the Bannatyne Campus Animal Care Committee,” University of Manitoba (approved protocol # 15066; 2017).

## Author Contributions

HM designed the research and experiments, supervised the research, and wrote the manuscript. SS, XJ, MR-B, MA, SC, SG, GC, and HM analyzed the data and co-wrote the manuscript. SS, XJ, MR-B, and MA performed the experiments. All authors have read and agreed to the published version of the manuscript.

## Conflict of Interest

The authors declare that the research was conducted in the absence of any commercial or financial relationships that could be construed as a potential conflict of interest.

## Publisher’s Note

All claims expressed in this article are solely those of the authors and do not necessarily represent those of their affiliated organizations, or those of the publisher, the editors and the reviewers. Any product that may be evaluated in this article, or claim that may be made by its manufacturer, is not guaranteed or endorsed by the publisher.

## Supplementary Material

Supplementary Figure 1 Expression of NCAM1 at P2, P5, and P7 in both *wt* and *nax* cerebellum by using Western blot; NCAM expression in *wt* and *nax* cerebellum is downregulated at P5 and P7.

Supplementary Figure 2 **(A–D)** FACS analysis of NEPH3 positive (Purkinje cells) and GLAST1 positive (Bergmann glia cells) from *nax* and *wt* mice cerebellum at the age of P2 **(A,B)** and P7 **(C,D)**. **(E)** We used cells incubated with secondary antibodies and DAPI only to set up the cut off levels for accurate sorting of the cells. The very low percentage of the cells were double positive (DP) which were excluded from mRNA extraction and the rest are double negative cells which are most of the cerebellar cells in both strains.

Supplementary Figure 3 The RNAseq data sets from the cerebellum at P5/P7 shows significant difference in expression of *Itgα3* and *Itgα5* between *nax* and *wt* littermate (***p* < 0.01, ****p* < 0.001).
