# Supplementary figures and images for: Upregulation of Neural Cell Adhesion Molecule 1 and Excessive Migration of Purkinje Cells in Cerebellar Cortex
Source: Front Neurosci. 2022 Jan 21;15:804402. doi: 10.3389/fnins.2021.804402 (PMC8814629; doi:10.3389/fnins.2021.804402)

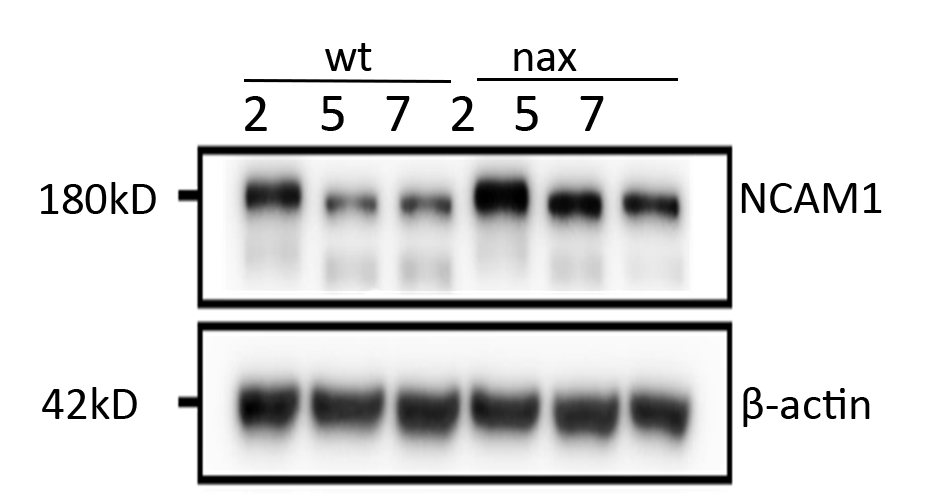

Supplement: Supplementary Figure 1 — Expression of NCAM1 at P2, P5, and P7 in both wt and nax cerebellum by using Western blot; NCAM expression in wt and nax cerebellum is downregulated at P5 and P7. [file Image_1.tif]

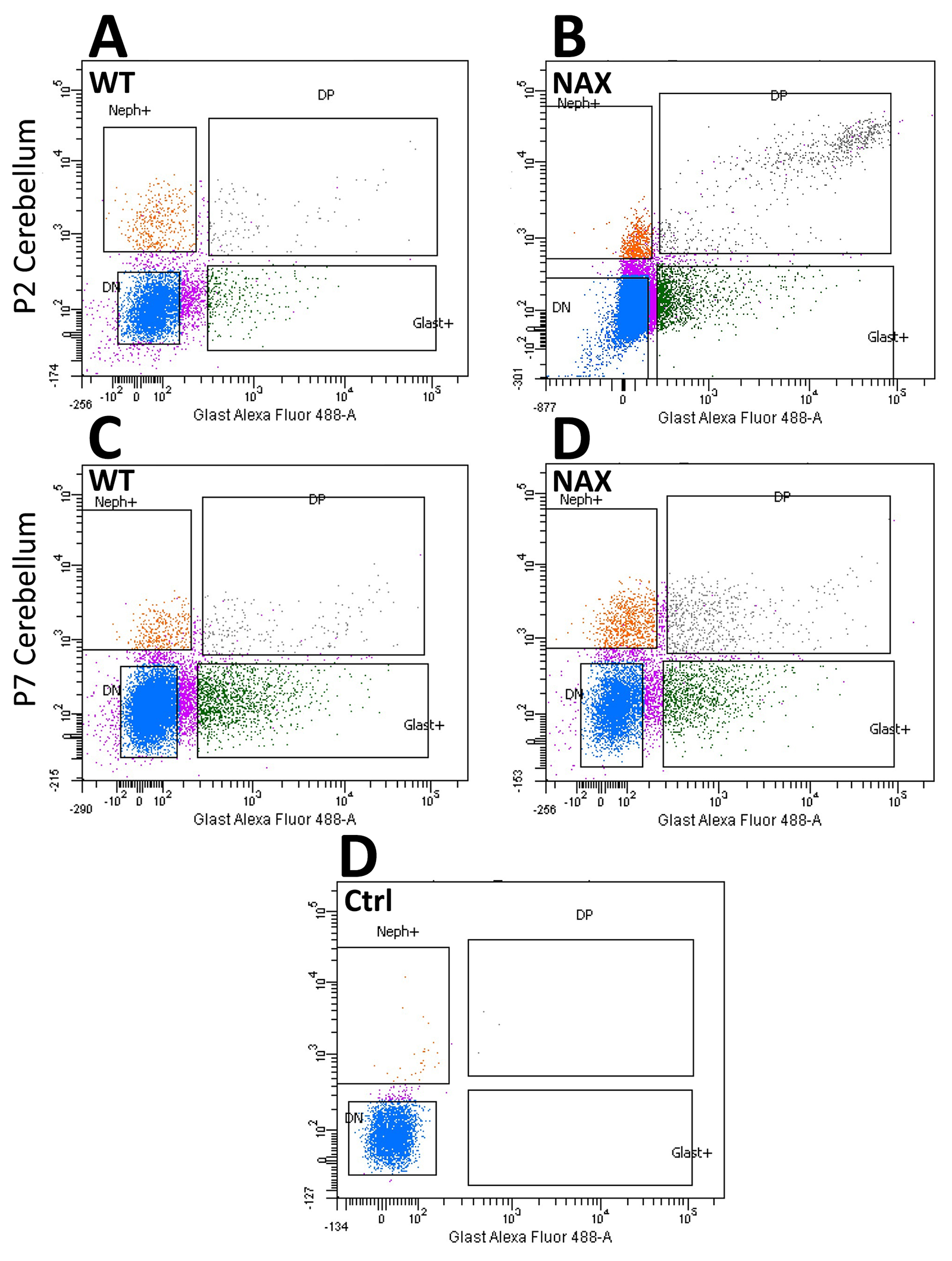

Supplement: Supplementary Figure 2 — (A–D) FACS analysis of NEPH3 positive (Purkinje cells) and GLAST1 positive (Bergmann glia cells) from nax and wt mice cerebellum at the age of P2 (A,B) and P7 (C,D). (E) We used cells incubated with secondary antibodies and DAPI only to set up the cut off levels for accurate sorting of the cells. The very low percentage of the cells were double positive (DP) which were excluded from mRNA extraction and the rest are double negative cells which are most of the cerebellar cells in both strains. [file Image_2.tif]

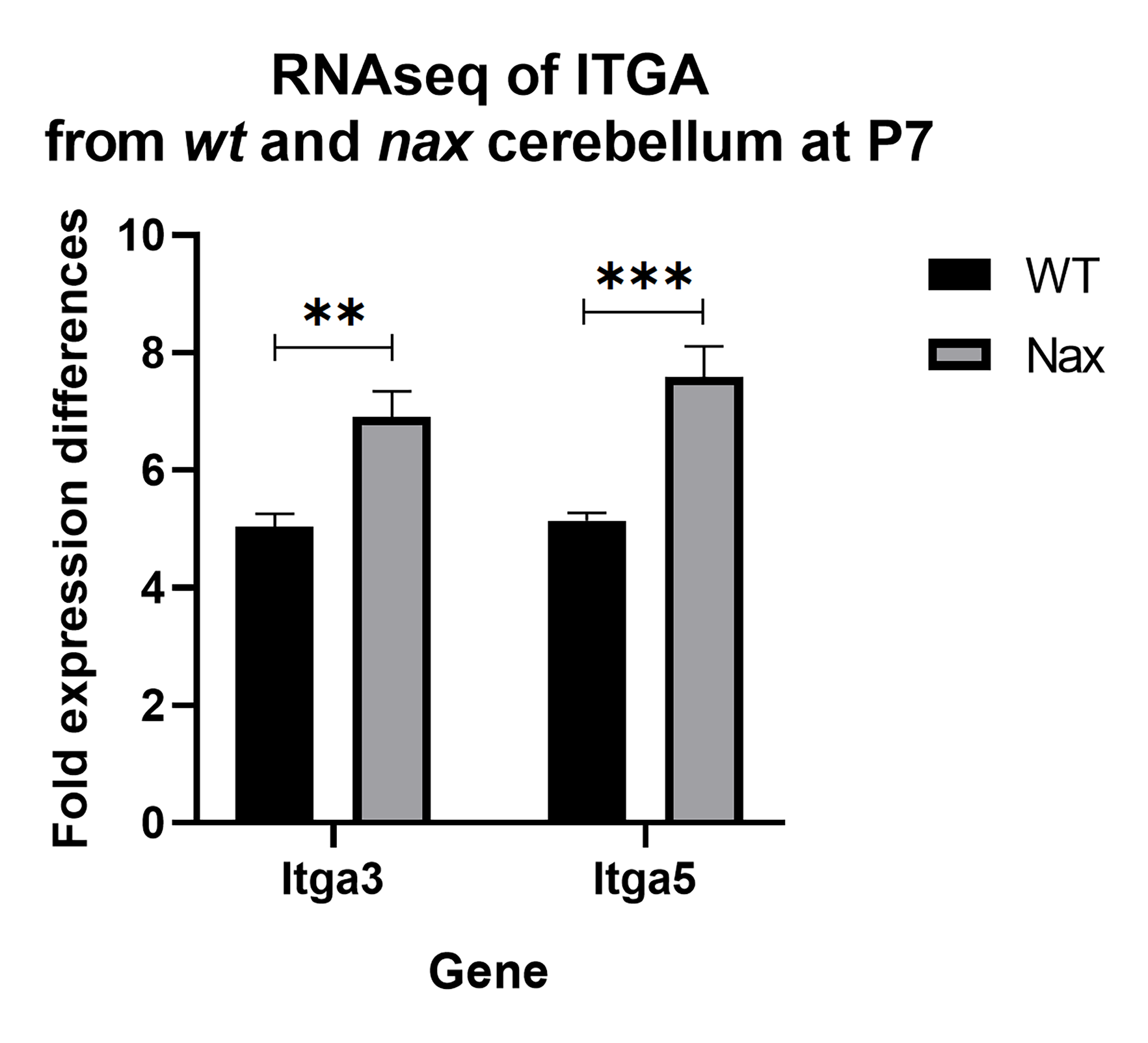

Supplement: Supplementary Figure 3 — The RNAseq data sets from the cerebellum at P5/P7 shows significant difference in expression of Itgα3 and Itgα5 between nax and wt littermate (**p < 0.01, ***p < 0.001). [file Image_3.TIF]
